# Caenorhabditis elegans Meets Microsporidia: The Nematode Killers from Paris

**DOI:** 10.1371/journal.pbio.1000005

**Published:** 2008-12-23

**Authors:** Jonathan Hodgkin, Frederick A Partridge

## Abstract

A newly discovered species of Microsporidia, which are obligate intracellular parasitic fungi, has been found able to infect the intestinal cells of the nematode*C. elegans* and subvert their cytoskeletal architecture.

Intracellular invasion is a great strategy for a microbial pathogen. By colonizing the cytoplasm or an intracellular compartment, the pathogen is shielded from many host defenses and gains privileged access to cellular nutrients. Intracellular pathogens are familiar in mammalian systems, in the form of bacteria such as Mycobacterium [1] and Listeria [2], and eukaryotic parasites such as Leishmania [3] and Trypanosoma cruzi [4]. The mechanisms whereby they get into cells and survive and multiply within them are central to their pathogenicity. Also, such pathogens often subvert host cellular machinery in interesting and informative ways. For example, the ability of Listeria cells to make “comet tails” of actin in order to propel themselves around cells has provided useful tools for investigating actin-based movement [5].

The invasion strategy is evidently ancient, and occurs even within bacteria themselves, in the form of bdellovibrios, which are bacteria able to live under the cell wall of other bacteria [6]. And the cytoplasm of all eukaryotic cells carries the inheritance of a primordial bacterial colonization, in the form of mitochondria and their derivatives. Many pathogens of invertebrates are also able to invade cells; one important instance is the penetration of insect gut cells by the malaria parasite during the mosquito phase of its lifecycle [7].

For nematodes, which constitute a vast and much studied invertebrate phylum, however, there have been few previous reports of pathogens capable of penetrating and multiplying within host cells. Nematodes are protected on the outside with an extremely tough and impermeable cuticle, which provides an excellent barrier against external attack. A few specialized parasites are able to drill through this armor plate and proliferate within the worm, such as the fungus Drechmeria coniospora [8] and the bacterium Pasteuria penetrans [9], but most diseases of nematodes involve attack from inside, via colonization of the alimentary tract, which is necessarily a more vulnerable tissue. Many free-living nematodes live by feeding on bacteria, so in a natural environment their guts are constantly exposed to a wide spectrum of potentially pathogenic microbes. Some of these can cause disease and ultimately death of infected worms, but the usual pattern of disease involves proliferation within the gut lumen, without any penetration of host cells.

A new pathogen, reported by Troemel et al. in this issue of *PLoS Biology* [10], significantly expands the roster of nematode diseases with the discovery of a microsporidian fungus able to form colonies and multiply inside the intestinal cells of the nematode Caenorhabditis elegans. This is likely to be extremely informative for studies of comparative pathogenicity, innate immunity mechanisms, and intestinal cell biology. It may also provide a convenient means of exploring microsporidian biology, much of which is still mysterious.

## The Nematode as a Model Host

**Figure 1:**
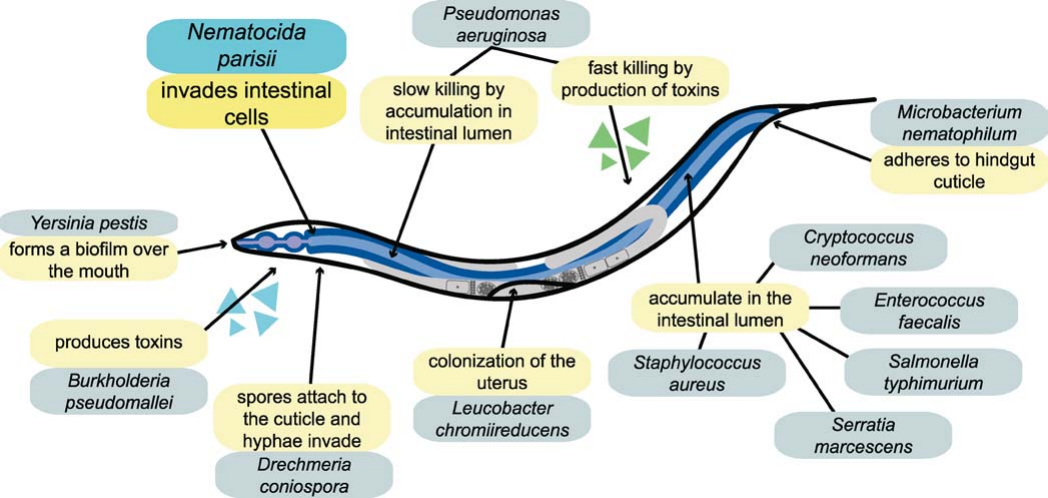
C. elegans and Its Enemies Diagram of C. elegans anatomy, indicating some of the pathogens under laboratory investigation and their modes of attack on the worm.

C. elegans has become an increasingly popular host for studying pathogenesis [11], following in the steps of Drosophila as a system for investigating conserved or novel innate immune mechanisms [12]. It is an established model organism, with numerous technical advantages, one of which is the simplicity of growing worms by feeding them with bacteria on agar plates. This lends itself to studying the effects of known pathogens, which can easily be tested for effects on the worm by exposing the worms to a lawn of the relevant bacterium [13,14]. Many bacterial and fungal species have now been shown to have toxic or pathogenic effects on the worm (Figure 1).

Various other pathogens of C. elegans have been discovered by chance contamination of laboratory cultures; one of these is the rectal pathogen Microbacterium nematophilum [15], and another is a recently described Leucobacter strain, which targets uterine tissues of the worm [16]. Each of these diseases provides expanded information on the vulnerabilities and defenses of C. elegans. This is significant both in its implications for innate immunity, and also for potentially providing novel ways of attacking nematodes themselves, many of which (though not the harmless C. elegans) are serious agents of disease in animals or plants.

## Discovery of a Natural Intracellular Parasite

**Figure 2:**
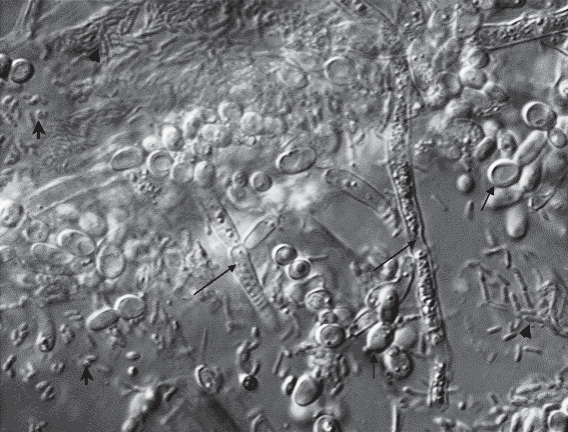
Complex Microflora Diverse bacterial and fungal cells found in association with a natural isolate of C. elegans in a decomposed apple, visualized by Nomarski microscopy. Long arrows mark filamentous fungi, short arrows mark yeast, barbed arrows mark round bacteria (cocci), and arrowheads mark rod-shaped bacteria. (Image courtesy of Marie-Anne Felix)

**Figure 3:**
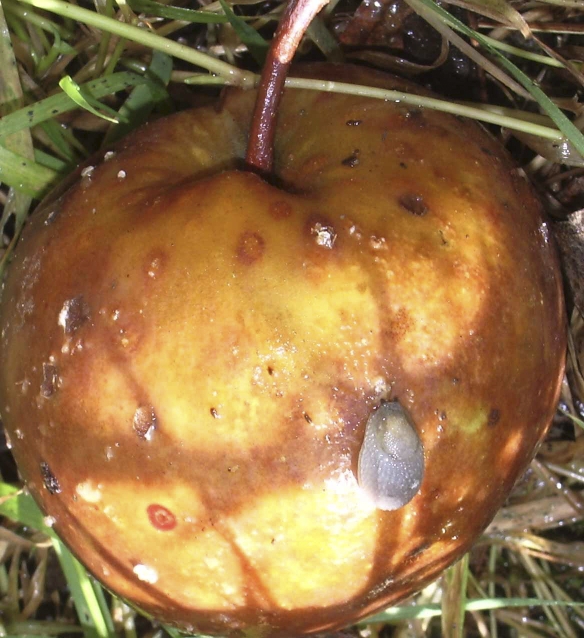
Where the Wild Worms Are Rotten apples and slugs, such as those shown here, are prime sources for finding new isolates of Caenorhabditis species, along with their natural pathogens. (Image courtesy of Marie-Anne Felix)

Wild isolates of C. elegans have not been mined as a source of novel pathogens until recently. This situation has changed with increasing interest in the natural ecology and evolution of Caenorhabditis species, so that much wider and more efficient global sampling of these nematodes is now going on [17]. Microbes isolated along with natural isolates also used to be ignored, because nematode samples would be routinely bleach-sterilized before establishing laboratory cultures. In the wild, Caenorhabditis species are frequently found in decaying vegetable matter, feeding on the rich and varied microbial blooms therein (Figure 2). Rotten apples are now known to be particularly productive source material (Figures 2 and 3).

The new pathogen was discovered from one sample of this type, a wild-caught C. elegans strain isolated from a compost pit in Franconville, outside Paris. The intestinal cells of these worms were observed to be full of small microbial rods, suggesting the presence of an intracellular pathogen. At least two different bacterial species could be detected by PCR in the original diseased isolate, but neither of these proved to be responsible for the intracellular rods. Treatment with varied antibiotics eventually eliminated the bacteria, but did not cure the worms of the intestinal infection. Further PCR experiments on the sick worms, using primers for both prokaryotic and eukaryotic rDNA, revealed the presence of sequences that could be assigned to the fungal phylum Microsporidia. The rDNA sequence was sufficiently diverged from its closest homolog to justify erection of a new microsporidian genus, Nematocida, and the pathogen was named Nematocida parisii.

## Microsporidian Infections

Microsporidia are extraordinary organisms [18]. Until recently it was unclear where they belonged in the tree of life, and they have the smallest genomes of all eukaryotes. Encephalitozoon cuniculi, a microsporidian pathogen of dogs, has a completely sequenced genome of only 2.9 Mb [19], and can be regarded as containing the minimal eukaryotic gene complement.

All microsporidia are obligate intracellular parasites, existing outside cells only in the form of tiny refractory spores. Each spore contains one or two nuclei and a characteristic structure called the polar tube, coiled up inside the spore [20]. The polar tube can be violently everted when the spore contacts a susceptible host cell, puncturing the host cell membrane and allowing injection of the nuclei and other spore contents. This invasion initiates the intracellular phase of the lifecycle. Inside the cell, the microsporidian grows to form a multinuclear plasmodium, or “meront.” Eventually the meront begins to form spores, which escape from the host and can infect new cells. Many animals, including humans, can act as a host for one or more microsporidian species; infection is often damaging and can be lethal, particularly in immunocompromised patients.

Light and electron microscopy by Troemel et al. reveal that N. parisii infection of C. elegans has many characteristic microsporidian features, including development of irregularly shaped multinuclear meronts inside intestinal cells and the subsequent formation of thick-walled spores containing polar tubes. The spores can transmit the infection horizontally, because healthy worms can be infected by culturing on the same plate as infected worms that are producing spores. Vertical transmission (from parent to offspring) is known to occur in some microsporidian infections, but not in this case, because only intestinal cells appear to be colonized, and surface-sterilized eggs from an infected parent hatch to yield healthy uninfected larvae.

## The Pathogen Induces Cytoskeletal Changes

Spore density in the infected intestinal cells can reach very high levels, seeming to pack most of the cytoplasm, and the worms eventually die prematurely, presumably from intestinal dysfunction. However, they are able to tolerate the infection for some time before dying, while continuing to shed infectious spores. The spores must therefore be able to escape from the intestinal cells without lysing them. A clue to the mechanism involved comes from immunofluorescent examination of intermediate filaments in the terminal web that underlies the intestinal microvilli. This web normally forms a continuous sheet, but becomes conspicuously patchy in spore-containing infected worms. The damage to the intermediate filament mesh appears to be selective, because a marker for microvilli reveals little alteration. So the holes in the web may allow spores to escape, like rabbits through a fence. This seems to be a specific effect of N. parisii, because the terminal web does not become disrupted in worms suffering from other kinds of severe intestinal infection.

## Known Immunity Pathways Are Ineffective against Microsporidia

An increasing amount is known about the innate immune defenses of C. elegans, mostly from examining intestinal infections with bacteria such as pathogenic strains of Pseudomonas aeruginosa and Staphylococcus aureus [13,14]. Two important pathways that contribute to immunity are a p38 MAPK pathway and the DAF-2/DAF-16 insulin-like signaling pathway; mutations abrogating the first of these pathways result in hypersusceptibility to many bacterial infections of the gut [21]. However, *pmk-1* mutants, which are defective in the first pathway, show little alteration in susceptibility to N. parisii. Conversely, *daf-2* mutants, which up-regulate the second pathway and exhibit increased resistance to bacterial pathogens [22], do not exhibit an obvious increase in resistance to N. parisii. So either these pathways do not contribute to resistance against the microsporidia, or else the parasite has evolved ways of inhibiting these immune reactions. In either case, deeper explorations of host responses to N. parisii, and possible defenses against it, promise to be rewarding.

## Further Discoveries of Nematode Microsporidia

The authors examined other wild-caught Caenorhabditis strains to see if they too might harbor microsporidia, and indeed two other French strains of C. elegans, from locations 30 and 300 km away from the original discovery site, were found to carry apparently identical N. parisii infections. Moreover, an Indian isolate of the related nematode C. briggsae was observed to carry intestinal microsporidia with an rDNA sequence signature slightly diverged from N. parisii and probably defining a distinct species. Comparable microsporidian infections may prove to be widespread among free-living nematode species. Their presence may not always be obvious, especially if the effects are only debilitating rather than lethal. The ability of C. elegans to survive and reproduce while tolerating high levels of cellular infestation by N. parisii suggests that there may have been significant coevolution between host and parasite. More benign microsporidian infections of other nematode species might easily have gone unnoticed hitherto.

## A Platform for Examining Microsporidian–Host Interactions

The intracellular growth pattern, and the lack of influence by known innate immunity pathways, make this infection distinct from previous host–pathogen interactions that have been studied in C. elegans. The powerful set of experimental techniques available for C. elegans, including facile forward and reverse genetics, can now be brought to bear on the microsporidian infection. Obvious questions are whether the worm has any dedicated defenses that ameliorate the effects of the gut infestation, and how the parasite is able to recognize its nematode host, invade, and then manipulate the cell architecture of the intestinal cells.

Whether N. parisii itself will also be amenable to genetic manipulation remains to be seen, but C. elegans may prove to be a very convenient host for investigating microsporidian biology in general, because the worms are so easily grown under laboratory conditions (unlike most other microsporidian hosts). The intestinal epithelium targeted by N. parisii is made up of a single layer of large cells, which have been well characterized anatomically, genetically, and molecularly [23]; moreover they can be readily visualized by light microscopy in the living animal. It should become possible to track the entire life cycle of N. parisii at high resolution, and, as Troemel et al. point out, it may also be possible to use this system for discovering drugs active against microsporidian infection.

## What Else Is Out There?

The discovery of N. parisii introduces a new member of the “enemies list” for C. elegans. Continued sampling and careful examination of wild-caught Caenorhabditis strains will almost certainly yield further examples of unusual pathogens. For example, a recent Japanese isolate of C. elegans has been found to be chronically infected with a novel strain of Leucobacter, which has effects similar to those of Microbacterium nematophilum (J. H. and M.-A. Felix, unpublished results).

However, there is one kind of pathogen that is still conspicuously absent from nematode biology: viruses. No natural viral pathogen has yet been found for any nematode species, although it has been shown that C. elegans cells are capable of supporting some kinds of viral replication [24,25,26]. Perhaps further sampling of French rotten apples will finally reveal the first true nematode virus.
